# Audio-Only Telehealth Use Among Traditional Medicare Beneficiaries

**DOI:** 10.1001/jamahealthforum.2024.0442

**Published:** 2024-05-10

**Authors:** Jiani Yu, Yasin Civelek, Lawrence P. Casalino, Hye-Young Jung, Manyao Zhang, Reekarl Pierre, Dhruv Khullar

**Affiliations:** 1 Division of Health Policy and Economics, Department of Population Health Sciences, Weill Cornell Medicine, New York, New York; 2 Division of General Internal Medicine, Department of Medicine, Weill Cornell Medicine, New York, New York

## Abstract

This cross-sectional study analyzes how use of audio-only telehealth services by Medicare beneficiaries changed from 2020 to 2022 and assesses which patients would be most affected by policy reforms.

## Introduction

Medicare expanded telehealth coverage during the COVID-19 pandemic, including for audio-only visits. Audio-only visits may be accessible to individuals lacking reliable internet for video visits; however, some have argued that these visits may lead to poorer quality care, increased costs, and fraudulent claims.^1,2^ Except for audio-only mental health visits, Medicare reimbursement for audio-only visits will expire after December 2024. We analyzed how audio-only telehealth use has changed over time and which patients would be most affected by policy reforms.

## Methods

This cross-sectional study used 100% Medicare fee-for-service carrier file claims from 2020 to 2022 to examine evaluation and management (E&M) visits among traditional Medicare beneficiaries. The E&M claims (*Current Procedural Terminology* [*CPT*] codes 99201-99215) billed with telehealth *CPT* modifier codes 95, GT, and FR were considered audiovisual telehealth visits.^3^ Claims were considered audio-only visits if they used audio-specific *CPT* codes for E&M services (99441-99443, 98966-98968) or E&M codes plus an audio-specific modifier (93) (available from January 2022).^3^ All other claims were considered in-person visits. This study was deemed exempt from review by the institutional review board at Weill Cornell Medicine because it did not involve human participants and all data were deidentified. We followed the STROBE reporting guideline.

We performed paired 2-sided *t* tests and χ^2^ tests comparing characteristics of beneficiaries who received 1 or more audio-only visits vs those who received audiovisual telehealth visits but no audio-only visits, and those who received only in-person care. Individual-level characteristics were obtained from the Medicare Beneficiary Summary File and included age, sex, race and ethnicity, rurality, and dual eligibility for Medicare and Medicaid. We constructed hierarchical condition category (HCC) scores using Centers for Medicare & Medicaid Services’ risk adjustment software. Two-tailed *P* = .05 indicated statistical significance.

## Results

Among 34 729 587 Medicare beneficiaries, 15 462 645 (44.5%) were females and 19 266 937 (55.5%) were males (mean [SD] age, 72.7 [10.5] years). Between 2020 and 2022, telehealth visits as a proportion of all Medicare E&M visits decreased from 16.1% to 7.8%; among telehealth visits, audio-only visits decreased from 31.0% to 25.4% (Figure). Only 1% of audio-only visits used newly available audio-specific modifiers.

**Figure.**
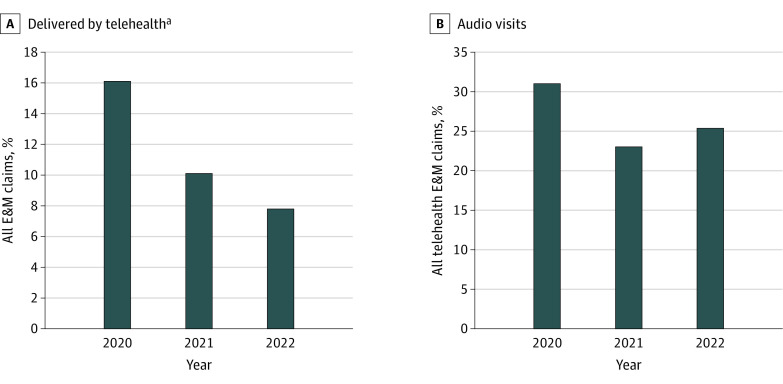
Changes in All Telehealth and Audio-Only Telehealth Evaluation and Management (E&M) Visits, 2020-2022 ^a^All telehealth claims included claims for both audio-only and audiovisual visits.

Compared with 72.5% of Medicare beneficiaries who received only in-person care in 2022, those who received at least 1 audio-only visit were more likely to be dually eligible (21.5% vs 13.5%), have medically complex conditions (HCC score, 1.7 vs 1.2), and be female (59.5% vs 55.0%), African American or Black (7.6% vs 7.0%), Hispanic (6.1% vs 4.7%), and less likely to reside in rural areas (1.5% vs 2.6%) (all *P* < .001) (Table). Compared with 17.5% of beneficiaries who received audiovisual telehealth visits, those with at least 1 audio-only visit were more likely to be dually eligible (21.5% vs 19.3%), have medically complex conditions (HCC score, 1.7 vs 1.5), be African American or Black (7.6% vs 7.1%), be older (mean [SD] age, 73.0 [11.1] vs 70.7 [11.1] years), and reside in rural areas (1.5% vs 1.2%) (all *P* < .001).

**Table.** Characteristics of Patients Receiving Audio-Only, Audiovisual, and In-Person E&M Visits in 2022

Characteristic	Type of E&M visit, No. (%)		
	≥1 Audio (n = 2 436 738)^a^	≥1 Audiovisual telehealth only (n = 4 251 960)^a^^,^^b^	In-person only (n = 17 625 713)^a^
Age, mean (SD), y	73.0 (11.1)	70.7 (11.1)	73.2 (10.1)
Sex			
Female	1 450 403 (59.5)	2 509 529 (59.0)	9 700 961 (55.0)
Male	986 335 (40.5)	1 742 431 (41.0)	7 924 752 (45.0
Dual eligibility	522 559 (21.5)	822 505 (19.3)	2 372 202 (13.5)
HCC score, mean (SD)^c^	1.7 (1.5)	1.5 (1.4)	1.2 (1.1)
Rural residence^d^	37 094 (1.5)	51 533 (1.2)	456 928 (2.6)
Race and ethnicity^e^			
African American or Black	185 882 (7.6)	301 051 (7.1)	1 232 734 (7.0)
American Indian or Alaska Native	19 070 (0.8)	14 578 (0.3)	83 958 (0.5)
Asian or Pacific Islander	80 892 (3.3)	165 412 (3.9)	464 078 (2.6)
Hispanic	148 054 (6.1)	259 669 (6.1)	822 349 (4.7)
Non-Hispanic White	1 927 422 (79.1)	3 350 722 (78.8)	14 477 691 (82.1)
Other^f^	22 291 (0.9)	41 775 (1.0)	134 905 (0.8)

Abbreviations: E&M, evaluation and management; HCC, hierarchical conditioning category.

^a^ All differences between the ≥1 audio and in-person only groups and the ≥1 audio and audiovisual telehealth only groups were statistically significant at *P* < .001.

^b^ Beneficiaries in this group did not have any audio-only visits.

^c^ Higher scores indicate worse health.

^d^ Defined as zip codes with a Rural-Urban Commuting Area classification of 10, based on the US Department of Agriculture 2010 Rural-Urban Commuting Area codes.

^e^ Categories were derived from the Research Triangle Institute’s race coding algorithm.

^f^ Includes individuals who reported more than 1 race.

## Discussion

In this cross-sectional study, telehealth visits as a proportion of all E&M visits and audio-only visits as a proportion of all telehealth visits decreased from 2020 to 2022. This may be partly due to uncertainty about continued payment for audio-only visits or about the quality and patient satisfaction associated with audio-only care.^2^ Audio-only visits might also have decreased as familiarity with audiovisual technologies increased.

Nevertheless, audio-only visits represented a quarter of all telehealth visits in 2022. Compared with beneficiaries who received only in-person care or audiovisual visits in addition to in-person care, those who received audio-only care were more likely to be African American or Black, have medically complex conditions, and be dually eligible. Restrictions on audio-only visits may disproportionately affect these beneficiaries. Prior research^4,5^ found that some older adults may prefer audio-only visits and that 41% of Medicare beneficiaries lack access to computers with high-speed internet.

A limitation of this study was that some audio-only visits may have been billed as audiovisual; our estimates may therefore represent a lower bound of audio-only use. Coding of audio-only visits may improve with greater use of audio-only modifiers. Given that audio-only visits may be an effective substitute for some audiovisual or in-person care,^6^ they could support access for individuals with limited digital connectivity.

## Notes

Supplement.
Data Sharing Statement
