# Supplementary material for: Audio-Only Telehealth Use Among Traditional Medicare Beneficiaries
Source: JAMA Health Forum. 2024 May 10;5(5):e240442. doi: 10.1001/jamahealthforum.2024.0442 (PMC11087829; doi:10.1001/jamahealthforum.2024.0442)
Supplement: Supplement. — Data Sharing Statement [file jamahealthforum-e240442-s001.pdf]

Data Sharing Statement

Yu. Audio-Only Telehealth Use Among Traditional Medicare Beneficiaries. *JAMA Health Forum*. Published May 10, 2024. doi:10.1001/jamahealthforum.2024.0442

Data
Data available: No
